# Supplementary material for: Comparative multiomics analysis of cell physiological state after culture in a basket bioreactor
Source: Sci Rep. 2022 Nov 23;12:20161. doi: 10.1038/s41598-022-24687-4 (PMC9686226; doi:10.1038/s41598-022-24687-4)
Supplement: Supplementary file 1 — Supplementary Information 1. [file 41598_2022_24687_MOESM1_ESM.zip › raw data/Transcriptomic raw data/6.Enrichment/2.KEGG/TE1vsTC1/ALL/src/csab04350.html]

csab04350


- csab:103237430

- Up regulated genes

103237430(1.35562492669487)

- csab:103237062

- Up regulated genes

103237062(1.44298253597733)

- csab:103246725

- Up regulated genes

103246725(2.36022198629957)

- csab:103246725

- Up regulated genes

103246725(2.36022198629957)

- csab:103246725

- Up regulated genes

103246725(2.36022198629957)

- csab:103245923

- Up regulated genes

103245923(1.06496862848235)

Close
